# A minimally invasive approach for atrial and ventricular sew-on epicardial lead placement

**DOI:** 10.1016/j.xjtc.2021.02.025

**Published:** 2021-03-01

**Authors:** Joseph R. Nellis, Mohammed K. Alsarraj, Jude S. Sauer, Jacob A. Klapper, Salim F. Idriss, Joseph W. Turek

**Affiliations:** a Department of Surgery, Duke University Hospitals, Durham, NC; b Duke Congenital Heart Surgery Research & Training Laboratory, Durham, NC; c LSI Solutions, Victor, NY; d Division of Cardiothoracic Surgery, Duke University Hospitals, Durham, NC; e Division of Cardiology and Electrophysiology, Duke University Hospitals, Durham, NC; f Pediatric and Congenital Heart Center, Duke University Hospitals, Durham, NC

**Figure:**
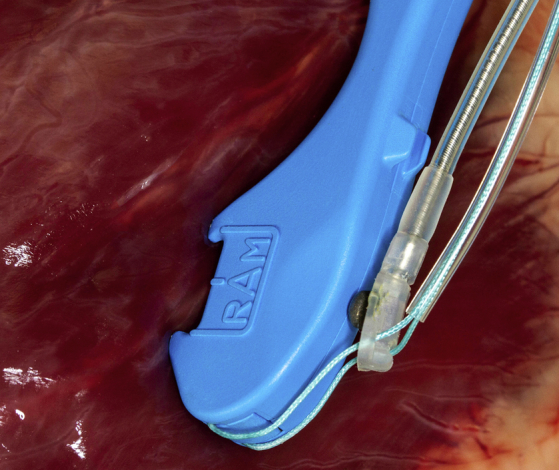
A protected dual-needle suturing device facilitates VATS sew-on epicardial lead placement.

**Central Message**

Atrial and ventricular VATS sew-on epicardial leads are possible. This approach provides traditional lead durability and avoids the risks associated with placing screw-in leads into the thin atrium.

See Commentaries on pages 249 and 251.

Permanent pacemaker (PPM) placement in pediatric patients is rare. Indications for permanent pacing in children include congenital and postsurgical atrioventricular block, symptomatic sick sinus syndrome, and select neuromuscular disorders.^1^^,^^2^ PPMs are traditionally placed using transvenous systems in adults and larger children. However, young children are often not suitable candidates due to their size or history of congenital cardiac surgery.

Epicardial lead placement is an alternative approach for patients who may not otherwise be candidates for transvenous systems. Traditionally, epicardial leads are placed through a median sternotomy during a larger operation, thoracotomy, partial sternotomy, or a subxiphoid incision.^3^^,^^4^ Screw-in epicardial leads exist; however, placement is typically limited to the ventricle and durability is reduced. Herein we describe a technique for placing atrial and ventricular sew-on epicardial leads through a minimally invasive video-assisted thoracoscopic surgery (VATS) approach in 5 children.

## Methods

### Patient Selection

In this series, VATS epicardial pacing was performed due to patient size, complex congenital heart disease, and the absence of additional procedures at the time of intervention. The preferred approach for pacing in infants and children at our institution, particularly in those with congenital heart defects, is dual-chamber epicardial pacing with a plan for future conversion to a transvenous system when the child is large enough if the cardiac anatomy is appropriate. Institutional review board waived consent for this study on May 19, 2020 (Pro00101549).

## Surgical Technique

**Figure 1:**
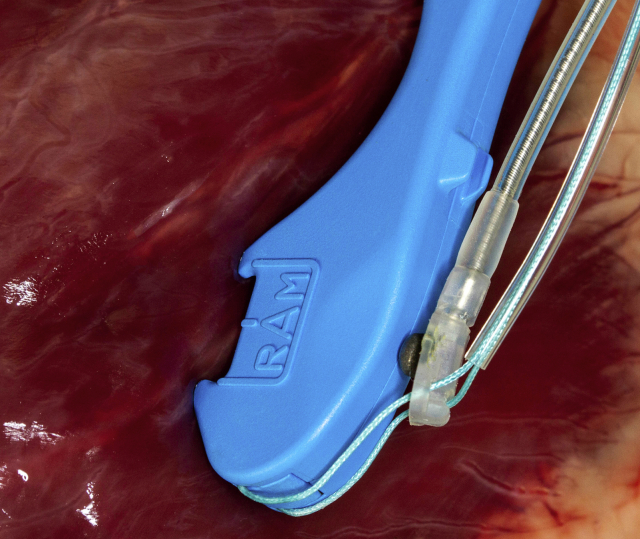
A protected dual-needle suturing device facilitates video-assisted thoracoscopic surgery sew-on epicardial lead placement.

After informed consent was obtained, patients were brought into the operating room, placed under general anesthesia and selectively intubated allowing for isolated single lung ventilation. The patient was then placed in right or left semilateral decubitus position depending on the laterality of the planned lead placement. Single lung ventilation was initiated and 3 5-mm ports were placed in the fifth, seventh, and ninth intercostal spaces along the left posterior axillary line or right posterior axillary line to provide retraction, instrumentation, and visualization, respectively (Video 1). The pericardium was opened sharply along the posterior aspect using endoscopic scissors, taking care to avoid the phrenic nerve. Steroid-eluting bipolar leads (Medtronic 4968; Medtronic, Minneapolis, Minn) were loaded onto a commercially available, dual-needle suturing device (Figure 1) (RAM; LSI Solutions, Victor, NY). The RAM device was used to simultaneously place 2 bites of a 3-0 suture in horizontal mattress fashion, securing the leads onto the ventricle or atrium. Sutures were then fastened with a titanium fastening device (Cor-Knot; LSI Solutions). The leads were tested, and if satisfactory, a tunnel was made from the generator pocket through the diaphragm under thoracoscopic visualization, and the opposite ends of the leads were passed to the generator pocket. If necessary, the generator was exchanged, and the leads tested again. A single chest tube was then placed, and all incisions were closed in 3 layers. Patients were extubated in the operating room and recovered in the intensive care unit.

Pain scores represent the average patient reported pain (score, 1-10) based on nursing flow sheets during a 24-hour period. Total morphine equivalents per day were calculated using previously published conversion factors.^5^

## Results

Five patients underwent VATS sew-on epicardial lead placement between May and September 2018. Patient ages ranged from 9 to 11 years with a median weight of 31 kg (interquartile range, 28-45 kg) and 2 prior median sternotomies (Table E1). Four patients successfully underwent minimally invasive sew-on epicardial lead placement. One patient with hypoplastic left heart syndrome and sinus node dysfunction following staged palliation was converted to a minithoracotomy due to dense adhesions. Patients spent a median of 1 night in the intensive care unit and a total of 4 nights in the hospital (Table 1).

**Table 1:** Perioperative data for video-assisted thoracoscopic surgery (VATS) epicardial lead placement

Patient	Date of surgery	Procedure performed	Single lung ventilation time (min)	Operative time (min)	Nights in ICU	Nights in hospital	Average pain POD 1 (ME Req)	Average pain POD 3 (ME Req)	Average pain at D/c (ME Req)
1	5/11/18	VATS RA lead	143	203	1	3	3.2 (5)	0 (0)	
2	2/22/18	VATS LV lead placement with generator exchange	102	162	2	3	3.5 (47.5)	0.5 (0)	
3	9/20/18	VATS LA and LV lead placement with generator replacement	144	275	2	9	1.3 (4)	0 (0)	0 (0)
4	9/27/18	VATS to thoracotomy LA and LV lead placement with generator placement	311	355	1	4	3.8 (29.8)	3.8 (13.8)	2 (0)
5	9/27/18	VATS LA and LV lead placement with generator placement	35	159	1	4	4.3 (30)	0 (12)	0 (9)
Median			143	203	1	4	3.2 (23)	0.9 (5)	0.5 (2)

*ICU*, Intensive care unit; *POD*, postoperative day; *ME Req*, morphine equivalent required; *D/c*, discharge; *VATS*, video-assisted thoracoscopic surgery; *RA*, right atrium; *LV*, left ventricle; *LA*, left atrium.

At a median 417 days follow-up (interquartile range, 379-738 days), atrial and ventricular lead impedance and voltage remained stable (Table 2). No phrenic nerve injury, surgical site infections, or musculoskeletal deficits were noted at the time of follow-up.

**Table 2:** Lead data following video-assisted thoracoscopic surgery epicardial lead placement

Patient	Perioperative lead data					Lead data at follow-up				
	POD	Impedance (Ohms)		Threshold (V)		POD	Impedance (Ohms)		Threshold (V)	
		Atrial	Ventricle	Atria	Ventricle		Atrial	Ventricle	Atria	Ventricle
1	3	476	–	0.375	–	738	436	–	0.5	–
2	0	–	513	–	2.25	788	–	532	–	2.25
3	4	742	636	1.625	2.5	379	1069	611	1.125	2
4	0	790	974	1.5	1.8	316	570	399	1.625	2.5
5	1	912	722	1.25	1.375	417	608	532	0.75	1.5
Median		766	679	1.37	2.0	417	589	532	0.9	2.1

*POD*, Postoperative day; *V*, volts.

## Conclusions

For select patients requiring PPM placement who are not candidates for transvenous systems, VATS sew-on epicardial lead placement is a minimally invasive option. Performed through 3 5-mm port sites, this approach provides single- or dual-chamber pacing options without the need for a partial sternotomy or thoracotomy. Within this select group of patients, there are no contraindications that we have identified. The largest limitations to this approach are the size of the working field and a surgeon's ability to operate thoracoscopically. Early trends in our series suggest that this approach is not associated with increased transfusion requirements, lengths of stay, postoperative pain, or complications, and it may serve as a useful alternative to screw-in lead placement in select patients.

## Figures and Tables

**Table E1:** Descriptive characteristics of male (M) and female (F) patients undergoing video-assisted thoracoscopic surgery epicardial lead placement

Patient	Age (y)	Sex	Weight (kg)	Indications	Prior operations
1	9	F	59	Maternal lupus, CHB, pacemaker syndrome now requiring atrial pacing	PPM via MS 2008, generator replacement 2008, left thoracotomy 2011 due to RV lead fracture
2	9	F	45	DORV, interrupted aortic arch repair, CHB requiring PPM now with poor LV lead conduction	MS 2008, PPM via repeat MS 2008, generator replacement 2013, revision to dual-chamber pacing via MS after lead fracture 2014
3	6	F	19	Trisomy 21, AV canal repair, CHB requiring PPM, infected leads and generator requiring replacement	MS 2011, PPM 2011, pMS 2018 for lead fracture and generator exchange, redo pMS 2018 for lead and generator removal due to infection
4	11	F	31	HLHS, sinus node dysfunction, symptomatic bradycardia, and syncope requiring PPM	Norwood MS 2007, Glenn MS 2007, Fontan MS 2011
5	9	M	28	Congenital CHB with poor functional status and inability to complete exercise testing due to fatigue	None

*CHB*, Complete heart block; *PPM*, permanent pacemaker; *MS*, median sternotomy; *RV*, right ventricle; *DORV*, double outlet right ventricle; *LV*, left ventricle; *AV*, atrioventricular; *pMS*, partial median sternotomy; *HLHS*, hypoplastic left heart syndrome.

## Supplementary Data

Video 1 Placement of a left ventricular video-assisted thoracoscopic surgery sew-on epicardial lead in a 9-year-old girl with history of previously repair double outlet right ventricular and interrupted aortic arch, complicated by complete heart block requiring permanent pacemaker, who is now undergoing lead exchange following fracture. Video available at: https://www.jtcvs.org/article/S2666-2507(21)00177-2/fulltext.
