# Supplementary figures and images for: A minimally invasive approach for atrial and ventricular sew-on epicardial lead placement
Source: JTCVS Tech. 2021 Mar 1;7:245–8. doi: 10.1016/j.xjtc.2021.02.025 (PMC8311829; doi:10.1016/j.xjtc.2021.02.025)

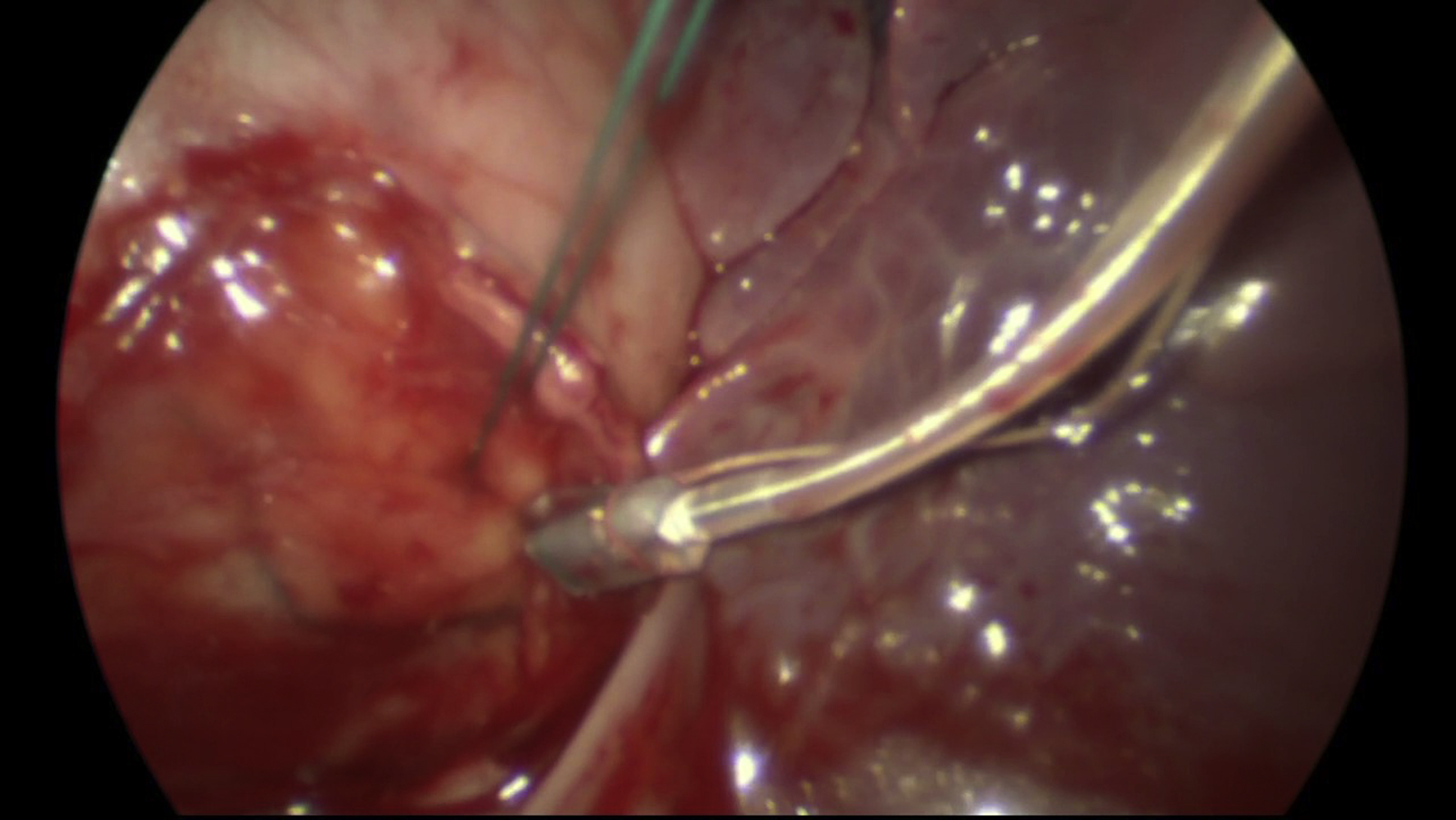

Supplement: Video 1 — Placement of a left ventricular video-assisted thoracoscopic surgery sew-on epicardial lead in a 9-year-old girl with history of previously repair double outlet right ventricular and interrupted aortic arch, complicated by complete heart block requiring permanent pacemaker, who is now undergoing lead exchange following fracture. Video available at: https://www.jtcvs.org/article/S2666-2507(21)00177-2/fulltext. [file fx2.jpg]
